# Nuclear translocation of mitochondrial ferredoxin reductase is regulated by AKT-mediated phosphorylation

**DOI:** 10.1042/BCJ20250303

**Published:** 2026-04-17

**Authors:** Ken-ichi Nakajima, Shakur Mohibi, Xinbin Chen, Jin Zhang

**Affiliations:** Department of Surgical and Radiological Sciences, University of California Davis School of Veterinary Medicine, CA, U.S.A.

**Keywords:** AKT, FDXR, NLS, Nuclear locolization, Phosphorylation

## Abstract

Ferredoxin reductase (FDXR) is the sole ferredoxin reductase in humans and plays an essential role for steroidogenesis and biosynthesis of heme and iron–sulfur cluster (ISC) by transferring electrons from NADPH to ISC-containing ferredoxin 1 (FDX1) and FDX2. In this study, we found that while FDXR is classified as a mitochondria-localized flavoprotein, it can be translocated into the nucleus, especially in response to various stress signals. Next, we identified a bipartite nuclear localization signal within amino acids 271–299 of FDXR, the disruption of which impairs its nuclear translocation. Further, we found that AKT can phosphorylate threonine 277 adjacent to the NLS in FDXR and subsequently enhances its nuclear translocation. Consistent with this, mutant FDXR(T277A), in which threonine 277 was substituted with alanine, impaired FDXR nuclear translocation. Together, our data provide evidence that the mitochondrial FDXR can be translocated to the nucleus, which is regulated by AKT-mediated phosphorylation, especially in response to cellular stress. Our data suggest that FDXR plays a role in the mitochondria–nucleus communication and stress responses.

## Introduction

Ferredoxin reductase (FDXR), also known as adrenodoxin reductase (ADX) [1–3], is a mitochondrial flavoprotein that plays a crucial role in the biosynthesis of steroids, heme, and iron–sulfur clusters (ISCs) [4]. FDXR catalyzes the transfer of electrons from nicotinamide adenine dinucleotide phosphate (NADPH) to two ferredoxin proteins, Ferredoxin 1 (FDX1) and FDX2 [5]. FDX1 subsequently donates electrons to cytochrome P450 enzymes involved in the synthesis of steroids, bile acids, and vitamins A and D [6,7]. On the other hand, FDX2 transfers electrons to enzymes involved in ISC biosynthesis, including IscU and other associated proteins [8–10]. Studies from our group and others have shown that total FDXR knockout mice and cancer cells are not viable [11,12], suggesting a critical role of FDXR in regulating cell growth. Additionally, germline mutations in the FDXR gene have been linked to various mitochondrial diseases in humans [13–15]. Further, we and others have demonstrated that FDXR deficiency leads to abnormal iron accumulation in mitochondria and disrupts lipid metabolism [16]. These observations indicate that FDXR plays critical roles in maintaining normal cellular function and metabolism via its mitochondria function.

Mitochondria play essential roles in cellular energy production and signal transduction. Increasing evidence indicates that certain mitochondrial proteins can translocate to the nucleus, where they influence gene expression and modulate cellular function. This process, known as mitochondria retrograde signaling, enables mitochondria to convey their functional state to the nucleus [17,18]. For example, nuclear translocation of mitochondrial protein can be activated in response to mitochondrial stress or dysfunction, such as impaired energy metabolism or oxidative damage, prompting changes in nuclear gene expression to help restore cellular homeostasis. Additionally, during mitochondrial injury or apoptosis, mitochondrial proteins may be released into the cytosol and inadvertently translocate into the nucleus, further impacting nuclear activities. Mitochondrial–nuclear communication has emerged as a critical mechanism by which cells adapt to various stresses. Dysregulation of mitochondria retrograde signaling has been linked to a wide range of diseases, including neurodegeneration, cancer, metabolic disorders, and aging [19,20].

To date, most studies focus on the role of FDXR in regulating metabolism and iron homeostasis in mitochondria. However, several lines of evidence suggest that FDXR may exert functions outside the mitochondria. First, according to the Entrez Gene database, FDXR is expressed in at least seven isoforms due to alternative splicing and the usage of different promoters. Sequence alignment indicated that some of isoforms, such as Isoform 7, lack the mitochondrial localization signal (MLS) and does not localize to mitochondria [21]. Importantly, although total FDXR-KO cells are non-viable, we were able to generate isoform-specific FDXR KO cells that only express Isoform 7, suggesting that FDXR exerts non-mitochondrial function [21]. Second, previous studies have shown that FDXR-deficiency disrupts mitochondria redox balance by interfering with electron transport and metabolic enzyme activity, leading to mitochondrial dysfunction [22,23]. Notably, mitochondrial dysfunction can activate retrograde signaling, which allows dysfunctional mitochondria to communicate with the nucleus to adapt the cell survival or metabolism or leads to global changes in gene expression [24–26]. Thus, it is possible that FDXR participates in retrograde signaling or exerts activities outside mitochondria.

To explore the biological function of FDXR, we found that FDXR undergoes nuclear translocation under various stress conditions. We also identified that FDXR contains a bipartite nuclear localization signal (NLS), which is also regulated by AKT via phosphorylation at threonine 277. Together, these findings uncover a novel regulatory mechanism of FDXR and FDXR undergo nuclear translocation.

## Results

### The FDXR protein is translocated into the nucleus in response to various stresses

**Figure 1:**
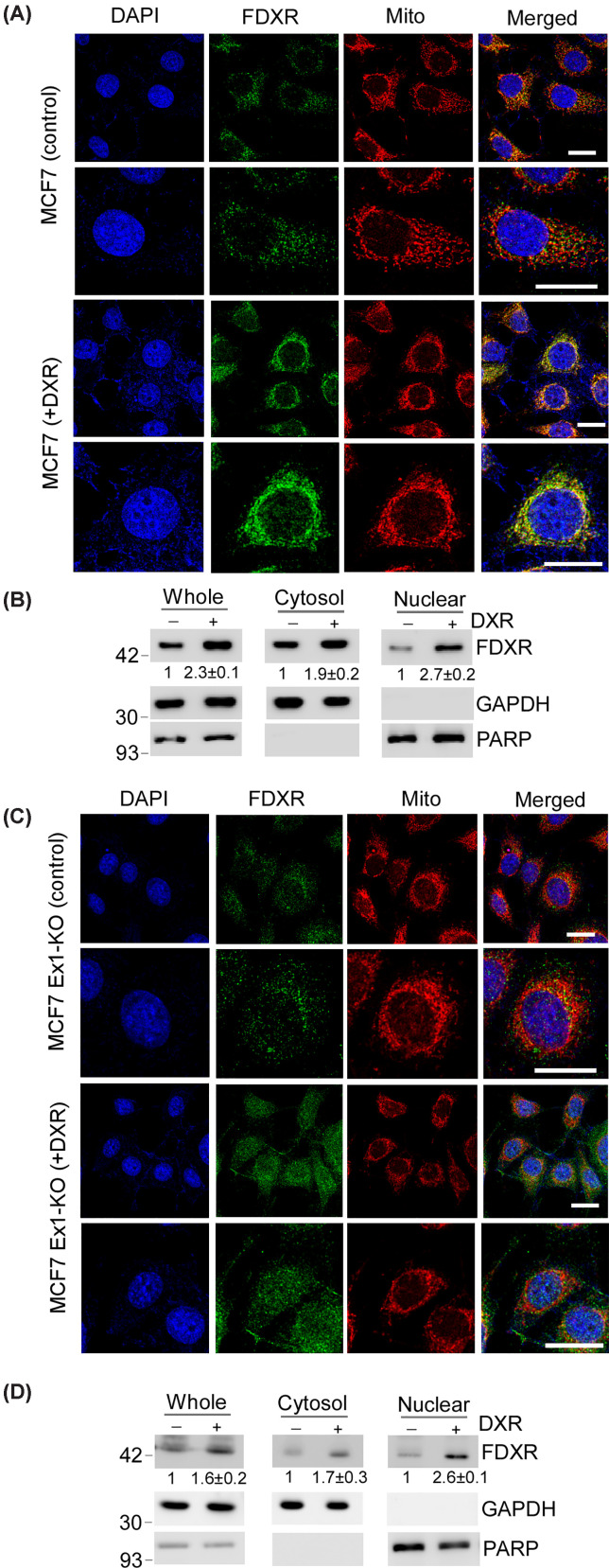
The FDXR protein is translocated into the nucleus in response to various stresses (**A**) MCF7 WT cells were treated with or without DXR, and endogenous FDXR was labeled by immunofluorescence staining with FDXR antibody. Mitochondria and nuclei were visualized by MitoTracker Deep Red and DAPI, respectively; scale bars: 20 μm. Representative images were shown, and the result was confirmed in other two replicates. (**B**) Subcellular fractionation assay was performed with MCF7 cells treated with or without DXR, followed by Western blot analysis to measure the level FDXR, GAPDH, and PARP protein. Representative images from triplicate experiments are shown and the relative fold changes were shown below each lane. (**C**) Exon 1-KO MCF7 were treated with or without DXR, followed by immunofluorescence assay as described in (A); scale bars: 20 μm. Representative images were shown, and the result was confirmed in other two replicates. (**D**) Ex1-KO MCF7 cells were mock-treated or treated with DXR, followed by subcellular fractionation assay to isolate the cytosol and nuclei. The expression of FDXR, GAPDH, and PARP was measured by Western blotting. Representative images from triplicate experiments were shown. The relative level of FDXR protein in control cells was arbitrarily set as 1.0 and the relative fold changes were shown as mean ± S.D. as below each lane.

According to Entrez gene database, FDXR is expressed as at least seven isoforms due to alternative splicing and the usage of two promoters. Sequence analysis showed that isoform 1 contains an intact MLS, while isoforms 4 and 7 have defects in their MLS (Supplementary Figure S1). Isoform 4 contains 31 aa insertions within its MLS whereas isoform 7 lacks the entire MLS and is not expected to be localized in the mitochondria (Supplementary Figure S1) [21]. Thus, it is possible that FDXR may exert an activity outside of mitochondria. To this end, MCF7 cells were treated with or without doxorubicin (DXR), a DNA damaging agent that induces DNA double-strand breaks by inhibiting topoisomerase II [27,28], followed by immunofluorescence staining with anti-FDXR, MitoTracker, and DAPI. Under non-stress conditions, FDXR showed a tubular/reticular staining pattern and is overlapped with MitoTracker staining, indicating that the majority of FDXR protein was associated with mitochondria (Figure 1A, MCF7 control, top two panels). Interestingly, upon DXR treatment, FDXR protein showed increased nuclear staining, suggesting its increased translocation to the nucleus (Figure 1A, MCF7 DXR, bottom two panels). To verify this, we sought to perform subcellular fractionation. Since FDXR is known to be cleaved at mitochondria and expressed as both a precursor and a mature protein [21,29], the relative abundance of each form was determined by Western blot analysis. We found that the FDXR antibody detected a single major polypeptide corresponding to the size of mature FDXR isoform, which was increased by DXR treatment and decreased by FDXR siRNA (Supplementary Figure S2), consistent with previous reports [21,29]. Next, subcellular fractionation was performed with MCF7 cells treated with or without DXR. We found that FDXR protein from whole cell lysates was increased by DXR (Figure 1B, left panel), consistent with previous finding that FDXR is induced by p53 in response to DNA damage [12,29,30]. Notably, FDXR was also detected in both cytosol and nucleus fractionations, which were further elevated following DXR treatment (Figure 1B, middle and right panels). As controls, we confirmed that GAPDH was present in the cytosol but not in the nucleus, whereas PARP was localized to the nucleus but absent from the cytosol (Figure 1B, middle and right panel). To further verify this, we examined whether FDXR is translocated to the nucleus in response to other stresses. We observed that the level of mature FDXR protein was elevated in nuclear fractionation lysates, following treatment with hydrogen peroxide (H_2_O_2_), Etoposide (ETP), and camptothecin (CPT) (Supplementary Figure 3A).

Previous study from our group and others indicated that FDXR isoforms 1, 4, and 7 are the major isoforms expressed in cells, accounting for approximately 70%, 10%, and 7% of total FDXR transcripts, respectively [21,31] (Supplementary Figure S3D). Notably, isoform 7 does not contain MLS and is not expected to be localized at mitochondria [21]. Thus, to determine whether various FDXR isoforms 1, 4, and 7 can be translocated in the nucleus, CRISPR–Cas9 technology was used to generate several isoform-specific FDXR-KO MCF7 cell lines, including Ex1-KO, Isoform 4-KO, and Isoform 7-KO (Supplementary Figure S3E) [21]. Ex1-KO cells only express isoform 7; Isoform 4-KO cells express isoforms 1 and 7; Isoform 7-KO cells express isoforms 1 and 4. To better visualize nuclear translocation of FDXR isoform 7, Ex1-KO cells were mock-treated or treated with DXR followed by immunofluorescence staining. Indeed, we found that the nuclear translocation of FDXR isoform 7 was increased in response to DXR (Figure 1C). Additionally, we found that FDXR isoform 7 in Ex1-KO cells exhibited a distinct localization pattern as compared to isoform 1. Isoform 7 does not colocalize well with MitoTracker (Figure 1C), consistent with the notion that isoform 7 lacks a MLS and is expressed outside of mitochondria. To verify this, subcellular fractionation assay was performed with Ex1-KO MCF7 cells treated with or without DXR. We found that isoform 7 was present in both cytosol and nuclear fractionation and the level of the nuclear isoform 7 was then further increased in response to DXR treatment (Figure 1D). Similarly, we found that in Isoform 4-KO and Isoform 7-KO MCF7 cells, FDXR proteins were also present in both cytosol and nuclear fractionation, which was further increased by DXR treatment (Supplementary Figure S3F and G). These findings suggest that FDXR protein is translocated to the nucleus in response to various stresses, which may play a critical role in maintaining cellular homeostasis by facilitating communication between the mitochondria and the nucleus.

DXR is known to induce mitochondrial dysfunction by disrupting the electron transport chain, especially complex I and II [32]. Thus, we examined whether the DRX treatment would alter localization of other mitochondrial proteins, such as succinate dehydrogenase subunit A (SDHA), the primary catalytic subunit of Complex II [33]. Specifically, cytosol, nuclear and mitochondria fractionations were isolated from MCF7 cells treated with or without DXR, followed by Western to detect FDXR and SDHA. We found that FDXR protein was present in both cytosol/mitochondria and nuclear fractionations, which was increased by DXR treatment (Supplementary Figure S4A, FDXR panel). In contrast, SDHA was detectable in cytosol/mitochondria but not nuclear fractionation (Supplementary Figure 4A, SDHA panel). Consistent with this, we also found that both FDXR and SDHA were present in mitochondrial fractionation (Supplementary Figure 4B, FDXR and SDHA panels). Additionally, FDXR but not SDHA was detectable in cytosol/nuclear fractionation (Supplementary Figure S4B, FDXR and SDHA panels). Thus, these data indicate that the DXR dose employed in this study has minimal impact on the localization of other mitochondrial proteins.

### Identification a bipartite nuclear localization signal in FDXR protein

**Figure 2:**
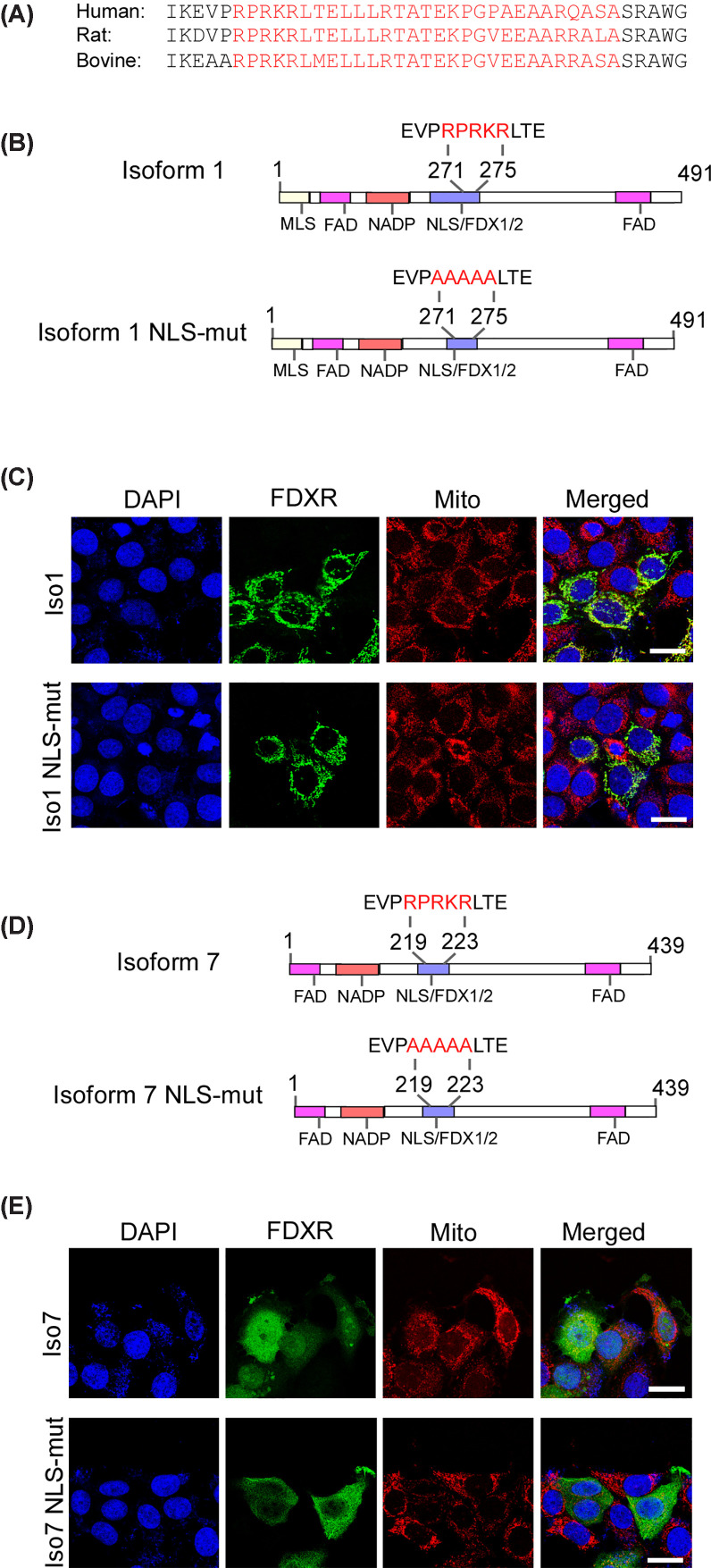
Identification of a bipartite nuclear localization signal in FDXR protein (**A**) A predicted bipartite NLS in found in FDXR protein and is conserved among human, rat, and bovine. The red letters indicated the bipartite NLS. (**B**) Diagrams of FDXR isoform 1 WT and NLS-mutant (NLS-mut). The positive amino acids (RPRKR) in the NLS were substituted by Alanine. (**C**) Immunofluorescence assay was performed with MCF7 cells transfected with 3xFlag-tagged WT FDXR isoform 1 or NLS-mut, followed by staining with anti-Flag, MitoTracker, and DAPI. The location of FDXR protein, mitochondria and nuclei were visualized by confocal microscope, respectively; scale bars: 20 μm. Representative images from triplicate experiments were shown. (**D**) Diagrams of FDXR isoform 7 WT and NLS-mutant (NLS-mut). The amino acids (RPRKR) in the NLS were substituted by Alanine. (**E**) MCF7 WT cells were transfected with 3xFlag-tagged FDXR isoform 7 WT or 3xFlag-tagged FDXR isoform 7 NLS-mut, followed by immunofluorescence assay. The FDXR protein, mitochondria, and nuclei were visualized by confocal microscope; scale bars: 20 μm. Representative images were shown, and the result was confirmed in other two replicates.

To investigate the mechanism how FDXR is translocated into the nucleus, we used the cNLS mapper tool (http://nls-mapper.iab.keio.ac.jp/cgi-bin/NLS_Mapper_form.cgi; last access on October 09, 2025) to identify a potential NLS. We found that one NLS is predicted at aa 271–299, which was highly conserved in other species, such as bovine and rat (Figure 2A) and classified as a bipartite NLS [34]. To verify that FDXR contains an NLS activity, we generated a mutant FDXR isoform 1 (called Iso1 NLS-Mut) in which aa 271–275 were substituted with alanines (Figure 2B). We found that the wild-type isoform 1 was localized in mitochondria and nucleus (Figure 2C, isoform 1). In contrast, Iso1 NLS-Mut failed to be translocated to the nucleus (Figure 2C, Iso1 NLS-Mut), indicating that the mutated region is required for nuclear localization. To verify the role of the NLS, we generated an NLS-mutant isoform 7 (Figure 2D). We found that wild-type isoform 7 exhibited a diffuse subcellular localization pattern in both the cytoplasm and nucleus (Figure 2E, Iso 7), which was distinct from the localization pattern observed for isoform 1. By contrast, the NLS-mutant isoform 7 showed only cytoplasmic localization and failed to be translocated to the nucleus, further supporting that the NLS is needed for FDXR to be translocated to the nucleus.

### Nuclear localization of FDXR is regulated by the AKT signaling

**Figure 3:**
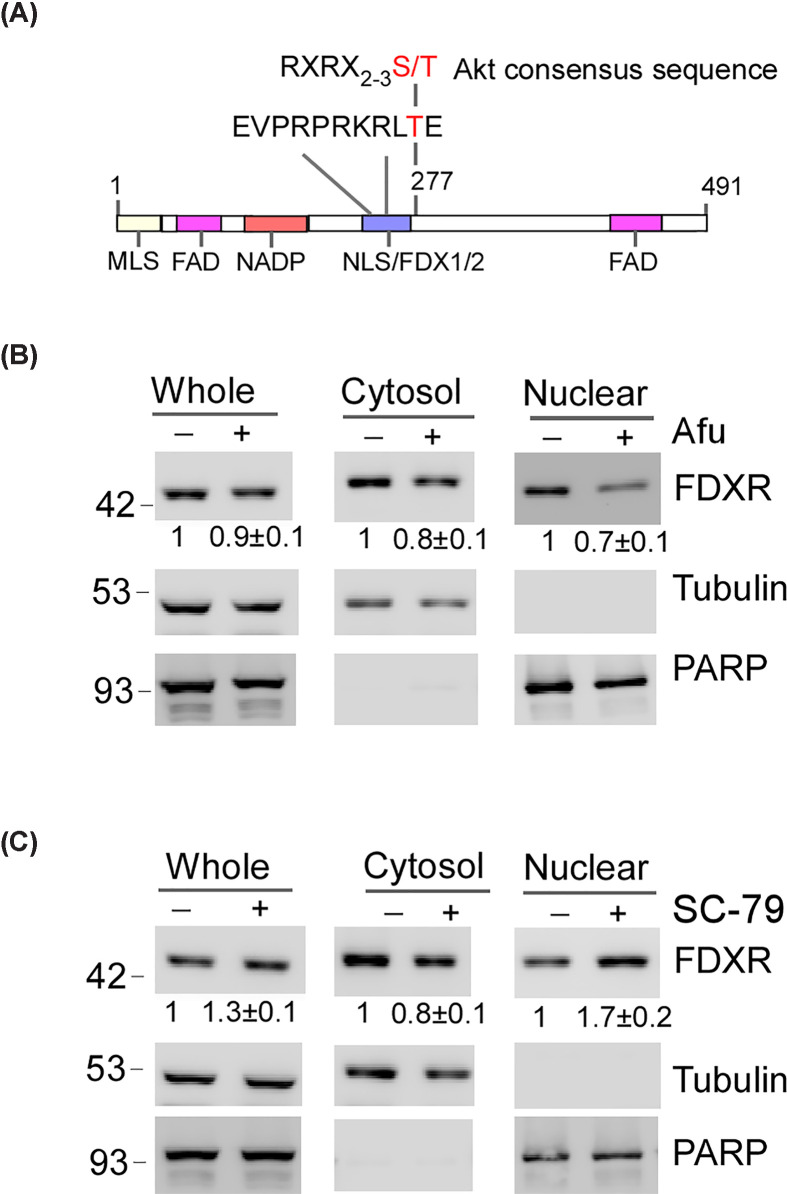
Nuclear localization of FDXR is regulated by the AKT signaling (**A**) Diagram of FDXR protein and the potential AKT-phosphorylation site within NLS. The AKT consensus sequence (RXRX_2-3_S/T) is also depicted. (**B**) MCF7 WT cells were treated with Afuresertib (5 μM) for 6 h, followed by subcellular fractionation assay to isolate whole cell lysates, cytosolic, and nuclear fractionation. The level of FDXR, tubulin, and PARP were measured by Western blotting. Representative images from triplicate experiments were shown and the relative fold changes were shown below each lane. (**C**) Nuclear localization of FDXR is enhanced by the AKT activator SC-79. MCF7 WT cells were treated with SC-79 (10 μM, 6 h), followed by subcellular fractionation assay as described in (B). Representative images from triplicate experiments were shown and the relative fold changes were shown below each lane.

Protein nuclear localization is tightly controlled by various mechanisms [35], such as phosphorylation and cytoplasmic retention through binding proteins, which allows for dynamic, stimulus-responsive transport of proteins into the nucleus to regulate gene expression and other cellular processes [36–38]. Thus, to explore how FDXR nuclear translocation is regulated, we sought to identify potential phosphorylation sites on the FDXR protein by using the PhosphoSitePlus database [39]. Indeed, FDXR can be phosphorylated at several sites including threonine 277 (T277). Interestingly, T277 is located in the NLS of FDXR, and its surrounding residues represent a consensus site for protein kinase AKT (Figure 3A). To this regard, SC-79 [40], an AKT activator, and Afuresertib (Afu) [41], an AKT inhibitor, were used. Specifically, to ensure that SC-79 and Afuresertib alter the AKT signaling pathway, phosphorylation of AKT at serine 473 and phosphorylation of GSK-3β at serine 9, were measured in MCF7 cells. We found that SC-79 leads to increased phosphorylation of AKT at serine 473 (Supplementary Figure S5A), consistent with a previous report [42]. We also found that Afuresertib resulted in decreased phosphorylation of GSK-3β at serine 9, known to be suppressed by Afuresertib after AKT inhibition [43,44]. Next, we sought to determine whether SC-79 and Afuresertib can modulate FDXR nuclear translocation by performing subcellular fractionation assay. We found that treatment with Afuresertib led to a reduction in the nuclear localization of FDXR protein, accompanied by a slight decrease in its cytosolic levels (Figure 3B). In contrast, treatment with SC-79 enhanced the nuclear localization of FDXR protein (Figure 3C). Together, these data suggest that nuclear localization of FDXR can be regulated by the AKT signaling.

### FDXR physically associates with AKT

**Figure 4:**
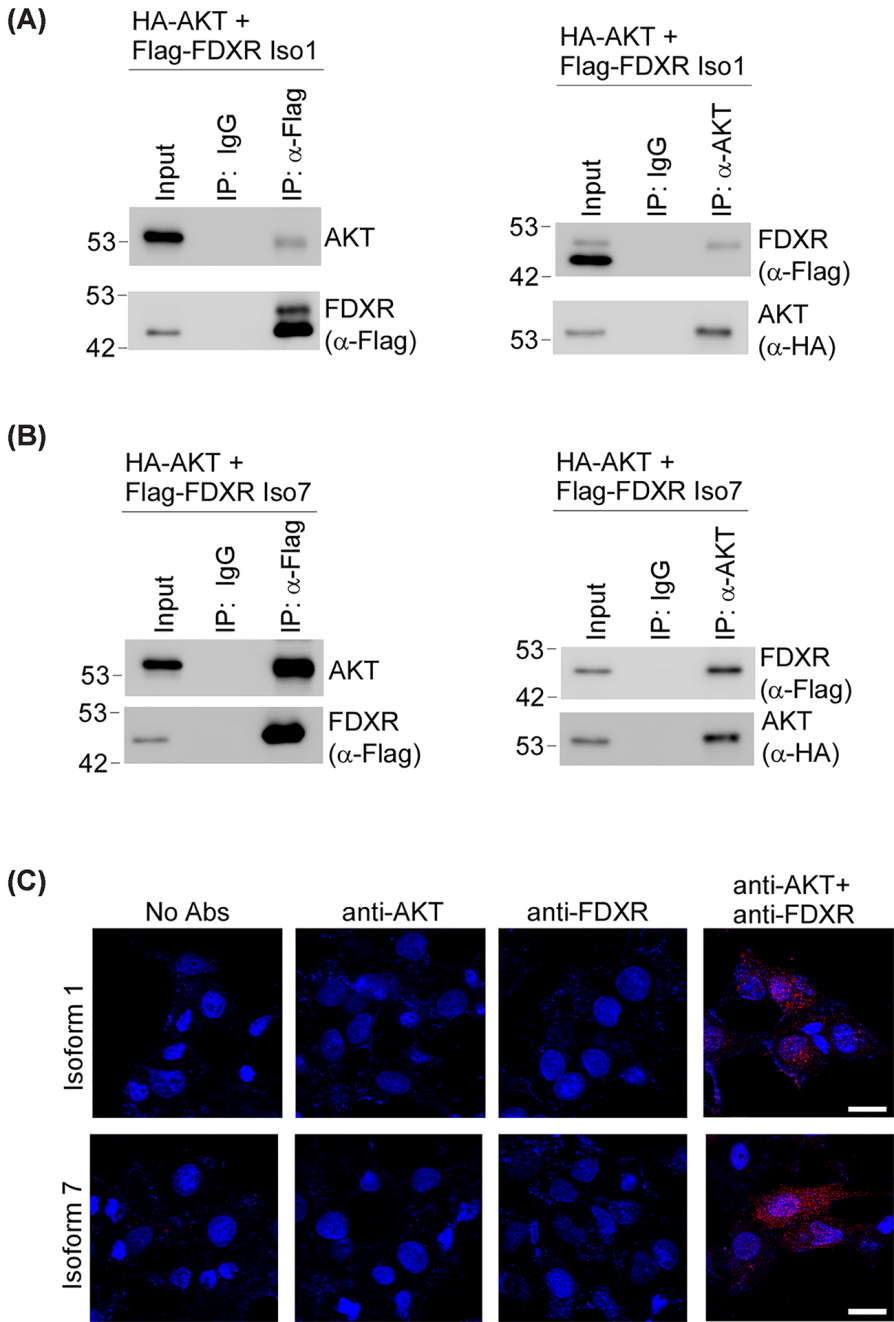
FDXR physically associates with AKT (**A**) 293T cells were co-transfected with 3xFlag-FDXR isoform 1 and HA-AKT1, followed by immunoprecipitation assay using anti-FLAG or anti-AKT. The immunocomplex were measured by Western blotting with indicated antibodies. Representative images from triplicate experiments were shown. (**B**) 293T cells were co-transfected with 3xFlag-FDXR isoform 7 and HA-AKT1. The cell lysates were then immunoprecipitated with anti-FLAG or anti-AKT, followed by Western blot to detect AKT and FDXR. Representative images from triplicate experiments were shown. (**C**) MCF7 WT cells were transfected with 3xFlag-tagged FDXR isoform 1 or 7, followed by PLA to visualize the association between FDXR and AKT; scale bars: 20 μm. Representative images were shown, and the result was confirmed in other two replicates.

To further investigate whether AKT phosphorylates FDXR, we determined whether AKT interacts with FDXR. To this end, reciprocal immunoprecipitation was performed with 293T cells transiently expressing Flag-tagged FDXR Iso1 and HA-tagged AKT. By using α-Flag to bring down FDXR, we found that AKT was present in the FDXR Iso1 immunocomplex (Figure 4A, left panel). In line with this, we also found that FDXR was present in the AKT immunocomplex (Figure 4A, right panel). Likewise, we found that AKT1 was present in the FDXR Iso7 immunocomplex (Figure 4B, left panel), and conversely, FDXR isoform 7 was present in the AKT immunocomplex (Figure 4B, right panel).

To verify that AKT interacts with FDXR, we employed *in situ* proximity ligation assay (PLA). Briefly, MCF7 cells were transfected with either FDXR Isoform 1 or Isoform 7, followed by PLA using antibodies against AKT and/or FDXR. We found that when probed with antibodies against FDXR and AKT, positive PLA signals were observed in cells expressing either FDXR isoform 1 or isoform 7 (Figure 4C, AKT + FDXR panel), indicating an interaction between AKT and FDXR Iso 1 or 7. Notably, PLA signals were also detected in the nucleus (Figure 4C), suggesting that the interaction between FDXR Iso 1/7 and AKT also occurs within the nuclear compartment. In contrast, no PLA signals were detected in negative controls, including the no-antibody control or when anti-FDXR or anti-AKT was used alone (Figure 4C, No Abs, anti-AKT and anti-FDXR panels). To further verify this, MCF7 cells stably expressing HA-tagged FDXR isoform 1 cells were also used for PLA assay. As expected, positive PLA signals were observed when probed with both anti-AKT and ani-HA (which recognizes HA-Tagged FDXR) (Supplementary Figure S6). Together, these data indicate that AKT interacts with FDXR Iso1 and 7.

### Phosphorylation of FDXR at threonine 277 is required for nuclear localization

**Figure 5:**
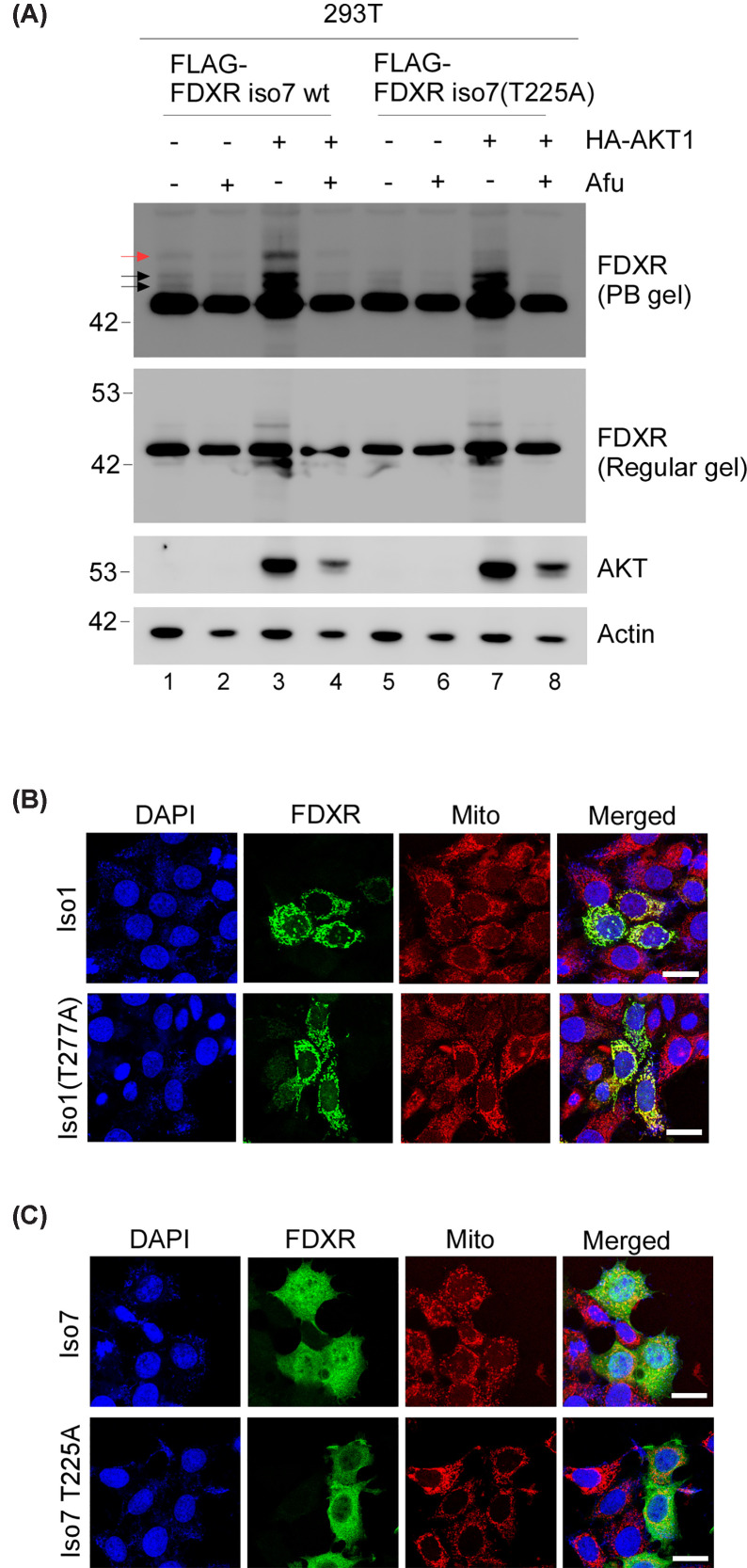
Phosphorylation of FDXR at threonine 277 is required for nuclear localization (**A**) 293T cells were co-transfected with 3xFlag-FDXR WT isoform 7 or T277A together with HA-AKT1, followed by treatment with or without Afuresertib. Total lysates were subjected to Phos-bind (PB) or regular acrylamide SDS–PAGE, followed by Western blotting to detect FDXR, AKT, or actin. Representative images from triplicate experiments were shown. (**B**) MCF7 WT cells were transfected with 3xFlag-tagged FDXR isoform 1 WT or T277A, followed by immunofluorescence assay with anti-Flag. Mitochondria and Nuclei were stained with MitoTracker Deep Red and DAPI, respectively; scale bars: 20 μm. Representative images were shown, and the result was confirmed in other two replicates. (**C**) MCF7 WT cells were transfected with 3xFlag-tagged FDXR isoform 7 WT or T225A, followed by immunofluorescence assay with anti-Flag. Mitochondria and Nuclei were stained with MitoTracker and DAPI, respectively; scale bars: 20 μm. Representative images were shown, and the result was confirmed in other two replicates.

As shown above, we found that nuclear FDXR localization is regulated by the AKT signaling (Figure 3), and FDXR and AKT physically interact (Figure 4). Therefore, we wanted to determine whether AKT phosphorylates FDXR at threonine 277. To test this, we employed Phos-bind acrylamide, a chemical reagent capable of distinguishing between phosphorylated and unphosphorylated forms of the same protein [45]. In the presence of divalent metal ions, such as Mn^2+^ or Zn^2+^, Phos-bind acrylamide forms a complex with phosphorylated residues, thereby reducing the electrophoretic mobility of these proteins, which can be detected by SDS–PAGE [46,47]. To this end, we constructed a mutant FDXR Iso7(T225A), in which threonine 225, corresponding to threonine 277 in isoform 1, was mutated to non-phosphorylatable alanine. Next, 293T cells were transfected with wild-type Iso7 or Iso7(T225A) along with or without HA-tagged AKT1 in the presence or absence of Afuresertib. The cell lysates were then analyzed by a regular or Phos-bind acrylamide SDS–PAGE, followed by Western blot to detect Isoform 7 or AKT. We found that Isoform 7 exhibited three distinct polypeptides as detected by Phos-bind acrylamide SDS–PAGE (Figure 5A, PB gel, labeled in arrows), as compared to that by regular SDS–PAGE (Figure 5A, regular gel). Moreover, these three polypeptides were decreased by treatment with Afuresertib but increased when co-expressed with AKT1 (Figure 5A, compare lanes 1 with 2–4). These data suggest that these three peptides are phosphorylated by AKT and that multiple AKT phosphorylation sites are present in FDXR. However, only one polypeptide (indicated by a red arrow) was absent from Iso7(T225A) (Figure 5A, compare lane 3 with 7), indicating that this polypeptide is phosphorylated at threonine 225. These data indicate that isoform 7 can be phosphorylated by AKT at T225, corresponding to T277 in isoform 1.

Next, we sought to determine whether phosphorylation of threonine 277 affects FDXR nuclear localization. To this end, MCF7 cells were transfected with a plasmid expressing wild-type Iso1 or mutant Iso1(T277A) in which threonine 277 was substituted with alanine. Immunofluorescence assay showed that wild-type Iso1 but not Iso1(T277A) was translocated to the nucleus (Figure 5B). To further verify this, the same experiment was performed using Iso7 and mutant Iso7(T225A). As expected, wild-type Iso7 was localized in both the cytoplasm and the nucleus (Figure 5C, top panels). By contrast, Iso7(T225A) was primarily localized at the cytoplasm (Figure 5C, bottom panel), which is similar to that exhibited by Iso7 NLS mut (Figure 2E). To further verify this, subcellular fractionation assays were performed. We found that wild-type FDXR Iso1 was present in both the cytosolic and nuclear fractions (Supplementary Figure S7A). In contrast, nuclear localization was impaired in cell lysates expressing the mutant FDXR(T277A) (Supplementary Figure S7A). Similarly, mutation of the threonine 225 in isoform 7 disrupted its nuclear localization (Supplementary Figure S7B). Together, these data suggest that phosphorylation of threonine 277 in FDXR by AKT promotes its nuclear localization.

## Discussion

FDXR has long been characterized as a mitochondrial flavoprotein and functions in electron transfer to ferredoxins to support mitochondrial P450-dependent reactions or synthesis of ISC protein. In the current study, we redefine this classical view by demonstrating that FDXR possesses a bipartite NLS and can be translocated to the nucleus, especially in response to various stress signals. We also found that the nuclear translocation of FDXR is modulated by AKT via phosphorylation of threonine 277. These findings suggest that FDXR may play a previously unrecognized role in the mitochondria–nucleus communication.

In this study, we found that AKT phosphorylates FDXR at threonine 277, promoting its nuclear translocation (Figures 4 and 5). In addition to FDXR, AKT has been found to promote nuclear localization of several other proteins via phosphorylation, such as hTERT [48], MDM2 [49], and mTORC1 [50]. Although a clear mechanism has not yet been elucidated, several possibilities exist. First, phosphorylation of FDXR by AKT may enhance the interaction of FDXR with the nuclear import machinery, such as importin α and β [51,52]. Second, AKT-mediated phosphorylation at T277 may regulate FDXR stability or subcellular distribution by modulating its association with other protein partners, thereby facilitating its nuclear accumulation. Third, since AKT is known to be expressed in the nucleus and modulates the activity of its nuclear substrates [53,54], it is possible that AKT phosphorylates FDXR within the nucleus to modulate its activity. Indeed, the Phos-bind acrylamide gel analysis indicated that in addition to threonine 277, AKT may phosphorylate other sites on FDXR (Figure 5A). Thus, further studies will be required to delineate the specific molecular mechanisms underlying this regulation.

The biological significance of nuclear localization of FDXR remains to be elucidated. It is possible that nuclear FDXR may participate in redox regulation of transcription factors, chromatin modifiers or DNA repair enzymes, all of which are highly sensitive to cellular redox balance. Previous studies showed that FDXR is one of the early induced genes in response to DNA damage [29,30,55]. Thus, nuclear FDXR might participate in the DNA damage response pathway, given that redox balance and electron transfer reactions are integral to DNA repair processes. Alternatively, FDXR may serve as a cofactor to modulate nuclear protein complexes in response to stress-induced signaling. Further, FDXR may contribute to the mitochondria-nucleus communication, a critical adaptive mechanism that coordinates metabolic and transcriptional responses under a stress. Such a role is consistent with emerging evidence that mitochondrial proteins can exert regulatory functions outside their primary organelle, thereby integrating energy metabolism with stress signaling pathways. Our data suggest that FDXR could be one of the mediators of this communication, possibly influencing the transcriptional landscape in response to oxidative or metabolic stresses. Defining the FDXR nuclear functions will be critical for understanding how FDXR contributes to cellular homeostasis and the integrated stress response.

In summary, our study uncovers a previously unrecognized role of FDXR by demonstrating that mitochondrial FDXR can be translocated to the nucleus, which is regulated by AKT-mediated phosphorylation, especially in response to cellular stresses. These findings point to future studies to identify the nuclear targets of FDXR, which would further our understating of the mitochondria–nucleus communication and elucidate how FDXR nuclear activity contributes to cellular homeostasis and stress responses.

## Materials and methods

### Reagents

Dulbecco’s modified minimal essential medium (DMEM) (Cat# 12100-061), ProLong Gold with DAPI (Cat# P36931), MitoTracker deep red (Cat# M22426), Mitochondria isolation kit for cultured cells (Cat# 89874), and fetal bovine serum (FBS) (Cat# 10437-028) were purchased from Thermo Fisher Scientific (Waltham, MA, U.S.A.). Penicillin (Cat# P93000-100.0) and streptomycin (Cat# 400-118P) were purchased from RPI Research Products (Mt. Prospect, IL, U.S.A.), and Genimi Bio-products (West Sacramento, CA, U.S.A.), respectively. Trypsin (Cat# 0458-250G) was purchased from VWR (Radnor, PA, U.S.A.). JetPRIME transfection reagent (Cat# 101000046) was purchased from Polyplus (Illkirch, France). WesternBright Sirius reagent (Cat# K-12043-D20) was purchased from Advansta Inc. (San Jose, CA, U.S.A.). DuoLink Proximity Ligation Assay kit (Cat# DUO92008, DUO90001, and DUO92005) and mouse monoclonal anti-FLAG M2 antibody (Cat# F1804) were purchased from Sigma–Aldrich (Burlington, MA, U.S.A.). FDXR antibody (Cat# sc-374436) and Tubulin antibody (Cat# sc-23950) were purchased from Santa Cruz Biotechnology (Dallas, TX., U.S.A.). Mouse monoclonal PARP1 antibody (Catalogue number 6639GR) was purchased from BD Bioscience (Franklin Lakes, NJ., U.S.A.). Rabbit monoclonal anti-GAPDH (Cat#:ber 2118), rabbit polyclonal anti-AKT (Cat# 9272), rabbit monoclonal anti-phospho AKT S473 (Cat# 4058), rabbit monoclonal anti-phospho GSK 3β S9 (Cat# 9323), and rabbit monoclonal anti-SDHA (Cat# 11998) were purchased from Cell Signaling Technology (Danvers, MA, U.S.A.). Mouse monoclonal anti-HA antibody (Cat# 901513) was purchased from BioLegends (San Diego, CA, U.S.A.). Protein A/G magnetic beads (Cat# HY-K0202) were purchased from MedChemExpress (Monmouth Junction, NJ, U.S.A.). Phos-bind acrylamide reagent (Cat# F4002) was purchased from APExBIO (Houston, TX, U.S.A.). All other chemicals and reagents were purchased from Sigma–Aldrich or Fisher Scientific unless otherwise specified.

### Cell culture

293T cells, MCF7 and its derivative cells, were maintained in DMEM supplemented with 10% FBS and penicillin–streptomycin at 37°C with air containing 5% carbon dioxide.

### Immunofluorescence microscopy

MCF7 WT cells were transfected with plasmid DNA using jetPRIME transfection reagent according to the manufacturer’s protocol. At 16–20 h after transfection, the cells were stained with 200 nM MitoTracker Deep Red for 30 min. The cells were fixed with 3.7% formaldehyde in phosphate buffered saline (PBS), permeabilized with 0.2% Triton X-100 in PBS, and blocked with 2% bovine serum albumin (BSA) in PBS. The cells were then stained with primary antibodies followed by fluorophore-conjugated secondary antibodies. The cells were mounted with ProLong Gold with DAPI and observed with Leica SP8 confocal microscope with a ×40 oil immersion objective. The final dilutions of the primary antibodies were 1:100 for anti-FDXR (Santa Cruz, mouse monoclonal), and 1:200 for anti-FLAG (Sigma, Mouse monoclonal).

### Immunoprecipitation

Cells were collected in an IP lysis buffer [50 mM Tris–HCl (pH7.5), 0.5% NP40, 1 mM EDTA, 150 mM NaCl, and protease inhibitor cocktail], sonicated, and centrifuged. The cell lysates were then incubated with an antibody and Protein A/G agarose beads at 4°C overnight (16–20 h) with gentle rotation. The beads were then washed 8 times with IP lysis buffer, and the immunocomplex were Western blotting described below.

### Western blotting

Cell lysates were separated by SDS–PAGE on 9–12% polyacrylamide gels and transferred to nitrocellulose membranes using a wet transfer system (Idea Scientific Company, Minneapolis, MN, U.S.A). Membranes were blocked in 2.5% non-fat dry milk in PBS-T buffer for 20 min at room temperature, followed by incubation with primary antibody overnight at 4°C. After washing with PBS-T, membranes were incubated with a designated secondary antibody for 3 h at room temperature. The membrane with then incubated with enhanced chemiluminescence (ECL), followed by protein detection using ChemiDoc Imaging Systems (Analytik jena). The final dilutions of the primary antibodies were: 1:2000 for anti-FDXR; 1:10,000 for anti-PARP; 1:5000 for anti-GAPDH; 1:1000 for anti-Tubulin;1:2000 for anti-FLAG; 1:1000 for anti-SDHA, 1:1000 for anti-phospho AKT S473, 1:1000 for anti-phospho GSK 3β S9, and 1:1000 for anti-AKT.

### RNA interference (RNAi)

MCF7 cells were transfected with 25 nM siRNA against FDXR using Lipofectamine RNAiMAX according to the manufacturer’s protocol. The sequences for scrambled siRNA was 5′-GCA GUG UCU CCA CGC ACU AdTdT-3′. The sequence for FDXR siRNA was 5′-CAC CAU UAA GGA GCU UCG G-3′.

### Analysis of phosphorylated protein with Phos-bind SDS–PAGE gel

Cell lysate was subjected to phos-bind SDS–PAGE that contained 50 μM of phos-bind acrylamide and 100 μM of MnCl_2_. After electrophoresis, proteins were transferred onto nitrocellulose membrane and subjected to Western blotting.

### Subcellular fractionation

Nuclear fractionation was carried out as previously described [56] with slight modifications. Briefly, cells were lysed in a hypotonic buffer (25 mM Tris–HCl (pH 7.4), 10 mM KCl, 2 mM MgCl_2_, 1 mM EGTA, 0.5 mM dithiothreitol, and protease inhibitor cocktail) and incubated on ice for 3–5 min. The cells were further incubated with 0.2% NP40 on ice for 5 min, followed by trypan blue staining to ensure plasma membrane lysis. Then, the mixture was centrifuged at 1000 × *g* at 4°C for 5 min to separate the nuclei (pellet) and cytoplasm (supernatant). The cytoplasmic fraction was further centrifuged at 15,000 × *g* for 5 min to remove debris, and the resulting supernatant was collected as the “cytosol/mitochondria” fraction. The nuclear fraction was suspended in an isotonic buffer [25 mM Tris–HCl (pH 7.4), 150 mM KCl, 2 mM MgCl_2_, 1 mM EGTA, 0.5 mM dithiothreitol, and protease inhibitor cocktail] containing 0.2% NP40, incubated on ice for 5–10 min, centrifuged at 1000 × *g* for 5 min, and the pellet was recovered. This same step was repeated one more time. Then the nuclear pellet was suspended in a RIPA buffer [25 mM Tris–HCl (pH 7.4), 150 mM NaCl, 0.1% SDS, 0.5% sodium deoxycholate, 1% NP40, and protease inhibitor cocktail] and incubated on ice for 20–30 min, followed by centrifugation at 2000 × *g* for 5 min. The resulting supernatant was collected as “nuclear” fraction.

Mitochondria and cytosol/nuclear fractionation was carried out using the Mitochondria isolation kit for cultured cells with a tight-fitting Dounce homogenizer according to the manufacturer’s protocol. Briefly, cells were resuspended in Mitochondria Isolation Reagent A and incubated on ice for 2 min. The cell suspension was transferred into Dounce homogenizer and homogenized on ice for ∼30 strokes. Mitochondria isolation reagent C was added to the homogenate, and the mixture was centrifuged at 700 × *g* for 10 min to remove nuclei and undisrupted cells. The supernatant was centrifuged at 3000 × *g* for 15 min. The resulting supernatant was collected as “cytosol/nuclear” fraction, and the pellet was collected as “mitochondria” fraction.

### Proximity ligation assay (PLA assay)

PLA assay was carried out by using DuoLink PLA assay kit according to the manufacturer’s protocol. Briefly, the cells were fixed with 3.7% formaldehyde in PBS, permeabilized with 0.2% Triton X-100 in PBS, and blocked with the blocking reagent supplied with the kit. The cells were incubated with primary antibodies at 4°C overnight (∼20 h). Next day, cells were first incubated with the PLUS and MINUS probes at 37°C for 1 h, and then with the DNA ligase at 37°C for 30 min, followed by DNA polymerase together with the fluorescent probe at 37°C for 100 min. The cells were mounted with ProLong Gold with DAPI and observed with Leica SP8 confocal microscope as described above.

## Supplementary Material

Supplementary Figures S1-S7

## Data Availability

All study data are included within the article.

## Open Access

Open access for this article was enabled through an open access agreement between the Biochemical Society/Portland Press and the University of California.

## Abbreviations

ADX: adrenodoxin reductase
CPT: camptothecin
DXR: doxorubicin
FDX1: ferredoxin 1
FDXR: ferredoxin reductase
ISC: iron–sulfur cluster
MLS: mitochondrial localization signal
SDHA: succinate dehydrogenase subunit A
PARP: Poly(ADP-ribose) polymerase-1
