# Supplementary material for: Nuclear translocation of mitochondrial ferredoxin reductase is regulated by AKT-mediated phosphorylation
Source: Biochem J. 2026 Apr 17;483(5):685–98. doi: 10.1042/BCJ20250303 (PMC13142927; doi:10.1042/BCJ20250303)
Supplement: Supplementary Figures S1-S7 [file BCJ-2025-0303_supp.pdf]

```
Iso 1  1     MASRCWRWWGWSAWPRTRLPPAGSTPSFCHH-------------------------------FSTQEKTPQICVVGSGPA  49
Iso 4  1     MASRCWRWWGWSAWPRTRLPPAGSTPTFGGSDEVRDPANAKALRNKRRRMQVRVKLGKFQLLLDIQEKTPQICVVGSGPA  80
Iso 7  1     ----------------------------------------------------------------MEDKDR----------  6

Iso 1  50    GFYTAQHLLKHPQAHVDIYEKQPVPFGLVRFGVAPDHPEVKNVINTFTQTAHSGRCAFWGNVEVGRDVTVPELREAYHAV  129
Iso 4  81    GFYTAQHLLKHPQAHVDIYEKQPVPFGLVRFGVAPDHPEVKNVINTFTQTAHSGRCAFWGNVEVGRDVTVPELREAYHAV  160
Iso 7  7     ---------EHPQAHVDIYEKQPVPFGLVRFGVAPDHPEVKNVINTFTQTAHSGRCAFWGNVEVGRDVTVPELREAYHAV  77

Iso 1  130   VLSYGAEDHRALEIPGEELPGVCSARAFVGWYNGLPENQELEPDLSCDTAVILGQGNVALDVARILLTPPEHLERTDITK  209
Iso 4  161   VLSYGAEDHRALEIPGEELPGVCSARAFVGWYNGLPENQELEPDLSCDTAVILGQGNVALDVARILLTPPEHLERTDITK  240
Iso 7  78    VLSYGAEDHRALEIPGEELPGVCSARAFVGWYNGLPENQELEPDLSCDTAVILGQGNVALDVARILLTPPEHLERTDITK  157

Iso 1  210   AALGVLRQSRVKTVWLVGRRGPLQVAFTIKELREMIQLPGARPILDPVDFLGLQDKIKEVPRPRKRLTELLLRTATEKPG  289
Iso 4  241   AALGVLRQSRVKTVWLVGRRGPLQVAFTIKELREMIQLPGARPILDPVDFLGLQDKIKEVPRPRKRLTELLLRTATEKPG  320
Iso 7  158   AALGVLRQSRVKTVWLVGRRGPLQVAFTIKELREMIQLPGARPILDPVDFLGLQDKIKEVPRPRKRLTELLLRTATEKPG  237

Iso 1  290   PAEAARQASASRAWGLRFFRSPQQVLPSPDGRRAAGVRLAVTRLEGVDEATRAVPTGDMEDLPCGLVLSSIGYKSRPVDP  369
Iso 4  321   PAEAARQASASRAWGLRFFRSPQQVLPSPDGRRAAGVRLAVTRLEGVDEATRAVPTGDMEDLPCGLVLSSIGYKSRPVDP  400
Iso 7  238   PAEAARQASASRAWGLRFFRSPQQVLPSPDGRRAAGVRLAVTRLEGVDEATRAVPTGDMEDLPCGLVLSSIGYKSRPVDP  317

Iso 1  370   SVPFDSKLGVIPNVEGRVMDVPGLYCSGWVKRGPTGVIATTMTDSFLTGQMLLQDLKAGLLPSGPRPGYAAIQALLSSRG  449
Iso 4  401   SVPFDSKLGVIPNVEGRVMDVPGLYCSGWVKRGPTGVIATTMTDSFLTGQMLLQDLKAGLLPSGPRPGYAAIQALLSSRG  480
Iso 7  318   SVPFDSKLGVIPNVEGRVMDVPGLYCSGWVKRGPTGVIATTMTDSFLTGQMLLQDLKAGLLPSGPRPGYAAIQALLSSRG  397

Iso 1  450   VRPVSFSDWEKLDAEEVARGQGTGKPREKLVDPQEMLRLLGH  491
Iso 4  481   VRPVSFSDWEKLDAEEVARGQGTGKPREKLVDPQEMLRLLGH  522
Iso 7  398   VRPVSFSDWEKLDAEEVARGQGTGKPREKLVDPQEMLRLLGH  439
```

Supplemental Figure1. Sequence alignments among FDXR Isoform 1, 4, and 7. The blue shaded area indicated the mitochondrial localization signal (aa 1-32) in Isoform 1. Isoform 4 contains 31 aa insertion in MLS. Isoform 7 is missing the entire MLS.

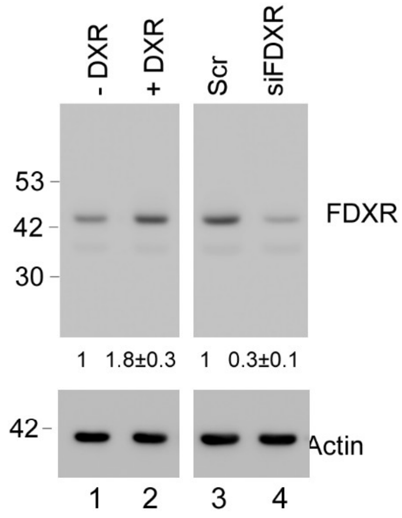

Supplemental Figure 2 Cell lysates were prepared from (Lane 1 and 2) MCF7 cells treated with or without 170 ng/ml of DXR (Lane 1 and 2) for 16 h, or MCF7 cells transfected with scramble siRNA (Scr) or siRNA against FDXR (siFDXR) (Lane 3 and 4), followed by Western blot to measure the level of FDXR and actin proteins. Representative images from triplicate experiments were shown. The relative level of FDXR protein in MCF7 control cells or MCF7 cells transfected with scrambled siRNA was arbitrarily set as 1.0 and the relative fold changes were shown as Mean±S.D. as below each lane.

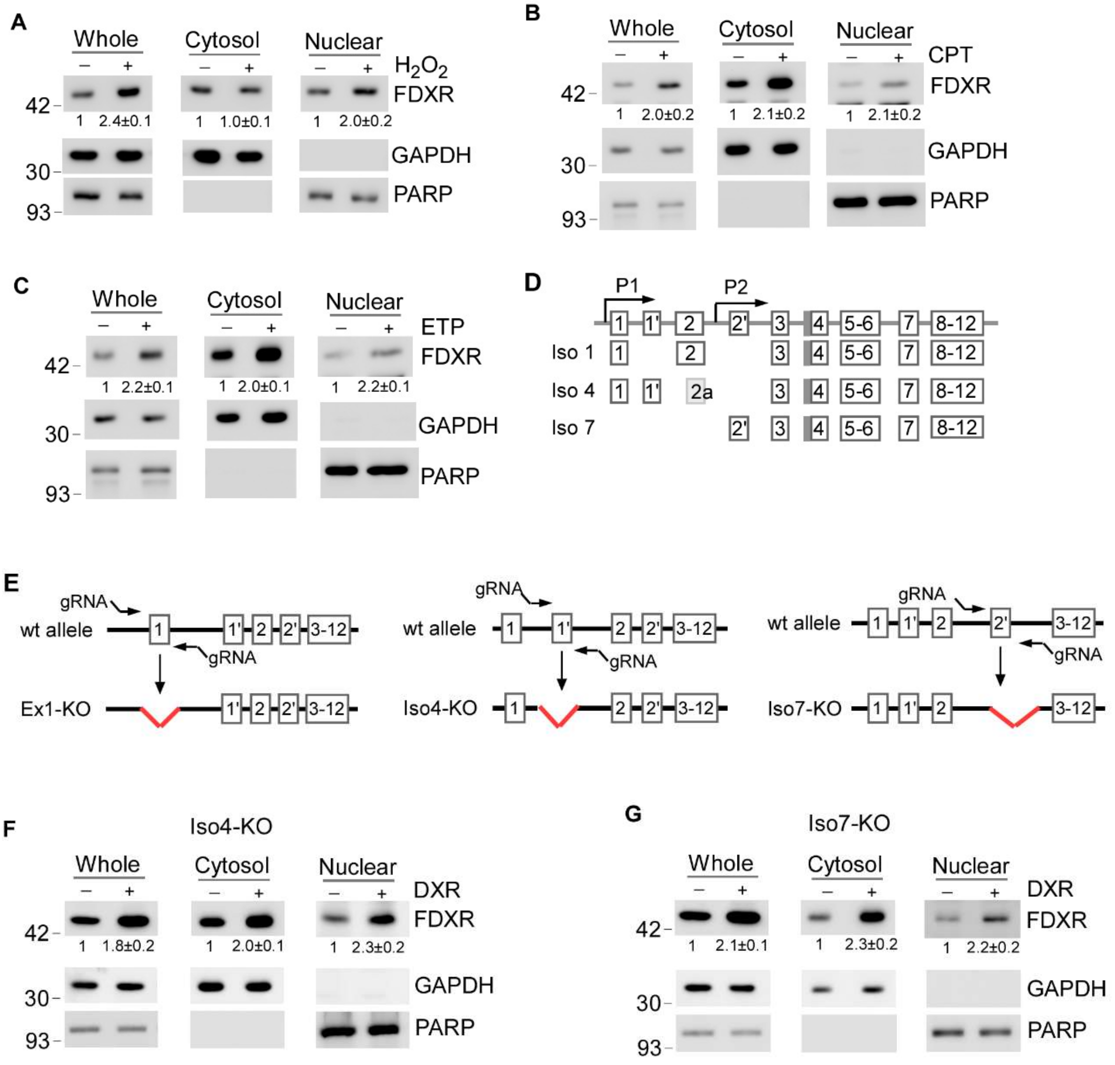

Supplemental Figure 3 (A) Subcellular cell fractionation assay was performed with MCF7 cells treated with or without 200 μM of H_2O_2 for 16 h, followed by western blot to measure the level of FDXR, GAPDH and PARP proteins. Representative images from triplicate experiments were shown. The relative level of FDXR protein in control cells was arbitrarily set as 1.0 and the relative fold changes were shown as Mean±S.D. as below each lane. (B) Subcellular cell fractionation assay was performed with MCF7 cells treated with or without 0.5 μM of camptothecin (CPT) for 14 h, followed by western blot to measure the level of FDXR, GAPDH and PARP proteins. Representative images from triplicate experiments were shown. The relative level of FDXR protein in control cells was arbitrarily set as 1.0 and the relative fold changes were shown as Mean±S.D. as below each lane. (C) Subcellular cell fractionation assay was performed with MCF7 cells treated with or without 10 μM of etoposide (ETP) for 14 h, followed by western blot to measure the level of FDXR, GAPDH and PARP proteins. Representative images from triplicate experiments were shown. The relative level of FDXR protein in control cells was arbitrarily set as 1.0 and the relative fold changes were shown as Mean±S.D. as below each lane. (D) Gene structure of FDXR isoform 1, 4, 7. (E) Schematic representation of the strategy to generate Ex1-KO, Iso4-KO and Iso7-KO MCF7 cell lines by CRISPR-Cas9. (F) Subcellular cell fractionation assay was performed with MCF7 Iso4-KO cells treated with or without doxorubicin, followed by western blot to measure the level of FDXR, GAPDH and PARP proteins. Red letters indicate the relative protein level of FDXR. Representative images from triplicate experiments were shown. The relative level of FDXR protein in control cells was arbitrarily set as 1.0 and the relative fold changes were shown as Mean±S.D. as below each lane. (G) Subcellular cell fractionation assay was performed with MCF7 Iso7-KO cells treated with or without 170 ng/ml of doxorubicin for 16 h, followed by western blot to measure the level of FDXR, GAPDH and PARP proteins. Representative images from triplicate experiments were shown. The relative level of FDXR protein in control cells was arbitrarily set as 1.0 and the relative fold changes were shown as Mean±S.D. as below each lane.

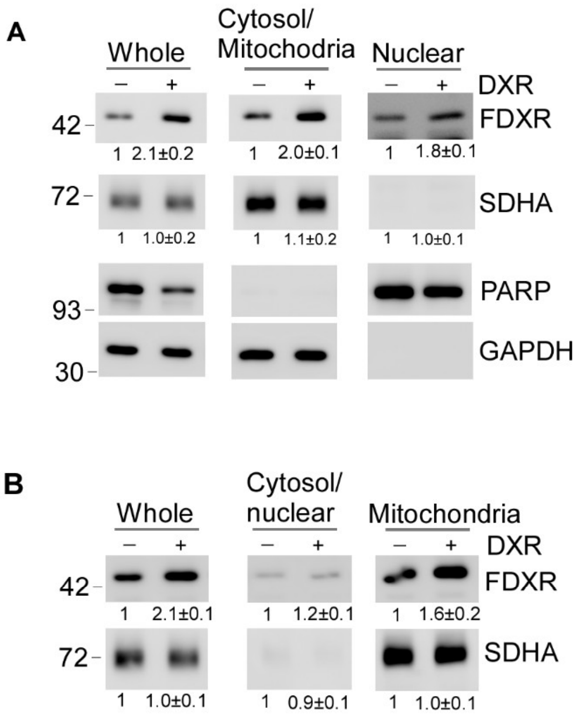

Supplemental Figure 4 (A) Nuclear fractionation assay was performed with MCF7 cells treated with or without 170 ng/ml of DXR for 16 h, followed by Western blot to measure the level of FDXR, SDHA, PARP, and GAPDH proteins. (B) Mitochondria fractionation assay was performed with MCF7 cells treated with or without DXR, followed by Western blot to measure the level of FDXR and SDHA proteins. Representative images from triplicate experiments were shown. The relative level of FDXR protein in control cells was arbitrarily set as 1.0 and the relative fold changes were shown as Mean±S.D. as below each lane.

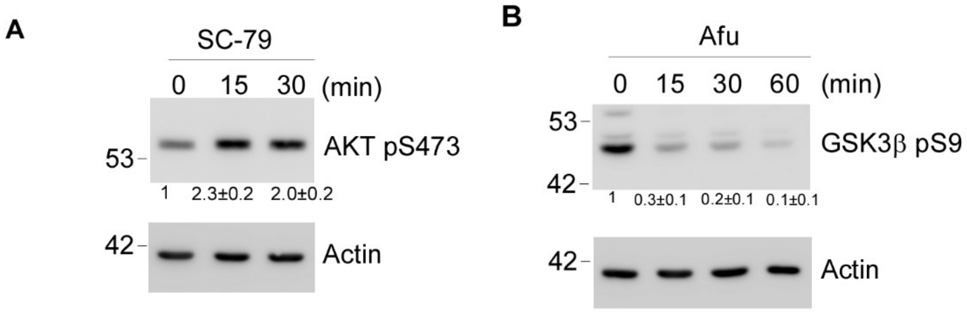

Supplemental Figure 5 (A) MCF7 cells were treated with or without SC-79 (10 μM) for 0, 15, 30 min, followed by Western blot to measure the level of phospho AKT (S473). phospho GSK 3β (S9), and actin proteins. Representative images were shown, and the result was confirmed in other 2 replicates. (B) MCF7 cells were treated with or without SC-79 (5 μM) for 0, 15, 30, 60 min, followed by Western blot to measure the level of phospho AKT (S473). phospho GSK 3β (S9), and actin proteins. Representative images from triplicate experiments were shown. The relative level of FDXR protein in control cells was arbitrarily set as 1.0 and the relative fold changes were shown as Mean±S.D. as below each lane.

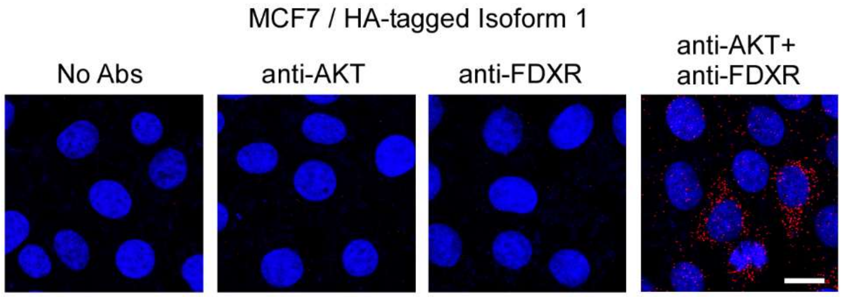

Supplemental Figure 6. PLA assay was performed with MCF7 cells expressing an HA-tagged FDXR to visualize the interaction between FDXR and AKT. The red dots indicated positive signals. Scale bar: 20 μM.

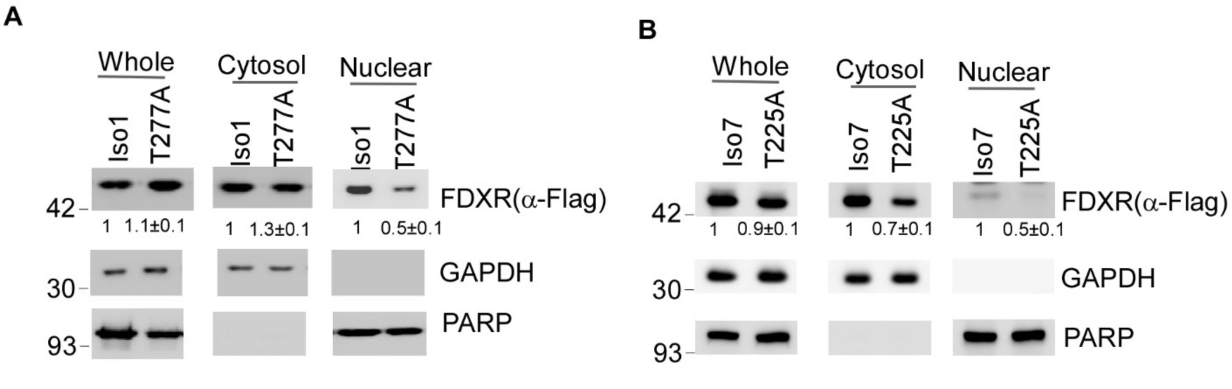

Supplemental Figure 7. (A) HCT116 cells were transfected with wild type FDXR Iso 1 or mutant FDXR(T277A), followed by cellular fractionation assay to isolate cytosol and nuclei. The cell lysates were subjected to western blot using antibodies against FDXR, GAPDH or PARP. Representative images from triplicate experiments were shown. The relative level of FDXR protein in control cells was arbitrarily set as 1.0 and the relative fold changes were shown as Mean±S.D. as below each lane. (B) MCF7 cells were transfected with wild type FDXR Iso 7 or mutant FDXR(T225A), followed by cellular fractionation assay to isolate cytosol and nuclei. The cell lysates were subjected to western blot using antibodies against FDXR, GAPDH or PARP. Representative images from triplicate experiments were shown. The relative level of FDXR protein in control cells was arbitrarily set as 1.0 and the relative fold changes were shown as Mean±S.D. as below each lane.
